# Real-Life Data From the Largest Pediatric Familial Mediterranean Fever Cohort

**DOI:** 10.3389/fped.2021.805919

**Published:** 2022-01-20

**Authors:** Kübra Öztürk, Taner Coşkuner, Esra Baglan, Hafize Emine Sönmez, Gülçin Otar Yener, Figen Çakmak, Fatma Gül Demirkan, Ayşe Tanatar, Serife Gül Karadag, Semanur Ozdel, Ferhat Demir, Mustafa Çakan, Nuray Aktay Ayaz, Betül Sözeri

**Affiliations:** ^1^Istanbul Medeniyet University, Göztepe Prof. Dr. Süleyman Yalçın City Hospital, Istanbul, Turkey; ^2^Department of Rheumatology, Umraniye Research and Training Hospital, University of Health Sciences, Istanbul, Turkey; ^3^Dr. Sami Ulus Child Health and Diseases Training and Research Hospital, Ankara, Turkey; ^4^Kocaeli University Faculty of Medicine, Izmit, Turkey; ^5^Sanliurfa Training and Research Hospital, Urfa, Turkey; ^6^Pediatric Rheumatology, Faculty of Medicine, Istanbul University, Istanbul, Turkey; ^7^Erzurum Regional Research and Training Hospital, Erzurum, Turkey; ^8^Zeynep Kamil Maternity and Childrens Hospital, Istanbul, Turkey

**Keywords:** familial Mediterranean fever, phenotype, genotype-phenotype correlation, pediatric, amyloidosis

## Abstract

Familial Mediterranean fever (FMF) is the most common monogenic autoinflammatory disease manifesting phenotypic heterogeneity. It is a clinically diagnosed disease supported by *MEditerranean FeVer (MEFV)* gene mutation analysis. However, the phenotype-genotype correlation is not yet established clearly. We aimed to determine the clinical findings, phenotype-genotype correlation, and treatment outcomes within a large pediatric FMF cohort. The medical charts of children with FMF who were diagnosed and followed up at the eight pediatric rheumatology units were reviewed retrospectively. All patients in the cohort were analyzed for sequence variants in exon 2,3,5 and 10 of the *MEFV* gene. Patients without any mutations or with polymorphisms including R202Q were excluded. A total of 3,454 children were involved in the study. The mean ± standard deviation of current age, age at symptom onset, and age at diagnosis were 12.1 ± 5.2, 5.1 ± 3.8, and 7.3 ± 4.0 years, respectively. Of 3,454 patients, 88.2% had abdominal pain, 86.7% had fever, 27.7% had arthritis, 20.2% had chest pain, 23% had myalgia, and 13.1% had erysipelas-like erythema. The most common *MEFV* mutation patterns were homozygous (32.5%) and heterozygous (29.9%) mutations of exon 10. Homozygous M694V was present in 969 patients (28.1%). Allele frequencies of common mutations were M694V (55.3%), M680I (11.3%), V726A (7.6%), and E148Q (7.2%). Children carrying homozygous or compound heterozygous exon 10 mutations had an earlier age of disease onset (4.6 vs. 5.6 years, *p* = 0.000) and a higher number of attacks per year (11.1 vs. 9.6, *p* = 0.001). Although 8% of the patients had a family history of amyloidosis, 0.3% (*n* = 11) had the presence of amyloidosis. M694V homozygosity was detected in nine patients who developed amyloidosis. Colchicine resistance was present in 4.2% of our patients. In this largest pediatric cohort reviewed and presented to date, patients with exon 10 mutations, particularly the M694V homozygous mutation, have been demonstrated earlier disease onset, annual attack count, and more frequent colchicine-resistant cases. Although E148Q is considered as a polymorphism in some populations, it was identified as a disease-causing mutation in our cohort. Secondary amyloidosis is still happening in adults however, it is extremely rare among children, presumably due to increased awareness, tight control, and the availability of anti-IL1 agents in colchicine-resistant cases.

## Introduction

Familial Mediterranean fever (FMF) is the most common inherited monogenic autoinflammatory disease manifesting with phenotypic heterogeneity (1). The disease results from the gain-of-function mutations located on the *MEFV* (*MEditerraneanFeVer*) gene. The *MEFV* gene encodes the protein pyrin which acts a part in the activation of the caspase-1 molecule and the production of interleukin (IL)-1-β (2, 3). To date, more than 350 sequence variations have been identified in the *MEFV* gene (4). A high acute phase response with self-limiting inflammatory attacks of recurrent fever, peritonitis, pleuritic, and arthritis is typical for FMF (5). It is a clinically diagnosed disease supported by *MEFV* gene mutation analysis especially for atypical cases (6).

Patients carrying homozygous exon 10 mutations such as M694V and M680I are known to have a severe phenotype, but heterozygous mutations for V726A and E148Q are associated with a milder disease course. There are many studies on this subject, but the phenotype-genotype correlation is not yet clearly identified. For instance, controversy continues regarding the potential pathogenic role of the E148Q (7–9). Another challenge is that, although FMF is autosomal recessive, about a quarter of patients do not have a mutation in the second allele but have typical clinical findings (10, 11).

Colchicine is the gold standard of treatment for FMF, and in addition to suppressing inflammation, it also prevents formation of amyloidosis. However, 5–10% of FMF patients do not respond despite adequate doses of colchicine (12). Anti-IL-1 therapy, anakinra, canakinumab, and rilonacept emerged as an alternative treatment for colchicine-resistant FMF patients (13).

In this study, we aimed to determine clinical findings, phenotype-genotype correlation, and treatment outcomes in a large pediatric FMF cohort in the light of new information in the literature.

## Patients and Methods

Medical files of 3,454 pediatric patients diagnosed with FMF according to the Turkish pediatric criteria (14) and followed up regularly in eight Pediatric Rheumatology Units were reviewed retrospectively. Demographic data, clinical features, and *MEFV* gene variant analysis were documented from medical charts. All patients in the cohort were analyzed for sequence variants in exon 2,3,5, and 10 of the *MEFV* gene. Patients without any mutations or with polymorphisms including R202Q were excluded from the cohort.

Alternative treatment options were also identified for resistant cases. Resistance to colchicine therapy was defined as ≥1 attack per month despite receiving the maximum tolerated dose for ≥6 months (13, 15). According to the recommendations (13), the colchicine dose was calculated from 1.2 mg/m^2^/day. The study has been approved by the institutional research ethics committee before was started and has been conducted by the principles outlined in the Helsinki Declaration. Written informed consents were taken from the legal guardians of the children.

### Statistical Analyses

Statistical analysis was performed by the SPSS software version 22 (SPSS Inc., Chicago, IL). The variables were investigated using visual (i.e., histograms and probability plots) and analytical methods (Kolmogorov-Smirnov/Shapiro-Wilk) to determine their distribution. Clinical and demographic characteristics were summarized by mean and standard deviation (SD) for continuous variables and count and percent for categorical variables. The categorical variables were compared with Chi-squared test. Bonferroni correction was used to adjust for multiple comparisons. Differences between independent samples were evaluated by the Student's *T*-test. A *p* < 0.05 was considered as statistically significant.

## Results

A total of 3,454 children (1,755 girls, 1,699 boys) were involved in the study. The mean ± SD of current age, age at symptom onset, and age at diagnosis were 12.1 ± 5.2, 5.1 ± 3.8, and 7.3 ± 4.0 years, respectively. The median (min-max) delay in diagnosis was 15 (0–230) months. Parental consanguinity was present in 30.5% of patients. One thousand nine hundred and eight patients (55.2%) had a family history of FMF. In addition, 8% of the patients had a family history of amyloidosis. It was determined that 97.3% of our patients met the Tel-Hashomer criteria (16) and 94.2% met the PRINTO/EuroFever 2019 criteria (17).

Of 3,454 patients, 88.2% had abdominal pain, 86.7% had fever, 27.7% had arthritis, 20.2% had chest pain, 23% had myalgia, 22.1% had exertional leg pain (ELP) and 13.1% had erysipelas-like erythema (ELE). The clinical findings of the patients are depicted in Table 1. When the patients were compared according to gender; arthritis and ELE were more frequent in females compared to males (*n* = 520 vs. *n* = 437, *p* = 0.01; *n* = 272 vs. *n* = 181, *p* < 0.001).

**Table 1:** The clinical findings of the familial Mediterranean fever patients (*n* = 3,454).

**Clinical findings**	***n* (%)**
Fever	2,994 (86.7%)
Abdominal pain	3,046 (88.2%)
Chest pain	699 (20.2%)
Pericarditis	20 (0.6%)
Arthritis	957 (27.7%)
Arthralgia	1,562 (45.2%)
Myalgia	793 (22.1%)
Erysipelas-Like erythema	453 (13.1%)
Exertional leg pain	764 (22.1%)
Protracted febrile myalgia	54 (1.6%)
Constipation	200 (5.8%)
Diarrhea	275 (8%)

The most common *MEFV* mutation patterns were homozygous (32.5%) and heterozygous (29.9%) exon 10 mutations. Homozygous M694V was present in 969 patients (28.1%) and allele frequencies of common mutations were M694V (*n* = 3,373, 55.3%), M680I (*n* = 782, 11.3%), V726A (*n* = 529, 7.6%), and E148Q (*n* = 503, 7.2%). The most common mutations of the cohort are summarized in Table 2. Genotype-phenotype analysis was performed by comparing the most common mutations with clinical findings. Accordingly, no relationship was found between different mutations and their association of fever and abdominal pain. However, chest pain was found to be common in patients with M694V/M680I (*p* < 0.001), M680I/M680I (*p* < 0.001), M694V/R761H (*p* < 0.001), and M680I/E148Q (*p* < 0.001) mutations. It was also shown that arthritis was more common in patients with M694V/M694V (*p* < 0.001) and R761H/- (*p* < 0.001) mutations. Mutations commonly seen with arthralgia, myalgia, and ELP, are shown in Table 3.

**Table 2:** The most common mutations in the familial Mediterranean fever cohort.

**Mutations**	***n* (%)**
M694V/M694V	969 (28.1)
M694V/M680I	283 (8.2)
M694V/V726A	235 (6.8)
M694V/E148Q	133 (3.9)
M680I/M680I	121 (3.5)
M680I/V726A	82 (2.4)
M694V/R761H	62 (1.8)
E148Q/E148Q	31 (0.9)
M680I/E148Q	30 (0.9)
E148Q/P369S	26 (0.8)
V726A/E148Q	25 (0.7)
P369S/R408Q	21 (0.6)
M694V/M694I	20 (0.6)
M680I/R761H	18 (0.5)
M694V/A744S	16 (0.5)
V726A/V726A	12 (0.3)
R761H/R761H	10 (0.3)
M694V/–	679 (19.7)
E148Q/–	216 (6.3)
V726A/–	135 (3.9)
M680I/–	122 (3.5)
R761H/–	35 (1.0)
A744S/–	30 (0.9)
K695R/–	17 (0.5)
P369S/–	11 (0.3)

**Table 3:** Comparison of the most common mutations and clinical findings in familial Mediterranean fever.

	**Abdominal pain**	**Fever**	**Chest pain**	**Arthritis**	**Arthralgia**	**Myalgia**	**ELP**	**Total (*n*)**
M694V/M694V	872 (90)	868 (89.6)	256 (26.4)	436 (45)^*^	501 (51.7)^*^	267 (27.6)^*^	293 (30.2)^*^	969
M694V/–	572 (84.2)	570 (83.9)	83 (12.2)	156 (18)	314 (46.2)	151 (22.2)	132 (19.4)	679
M694V/M680I	248 (87.6)	240 (84.8)	81 (28.6)^*^	71 (25.1)	111 (39.2)	46 (16.3)	59 (20.8)	283
M694V/V726A	210 (89.4)	215 (91.5)	52 (22.1)	42 (17.9)	90 (38.3)	41 (17.4)	36 (15.3)	235
E148Q/–	189 (87.5)	179 (82.9)	25 (11.6)	50 (23.1)	111 (51.4)^*^	61 (28.2)^*^	47 (21.8)	216
V726A/–	121 (89.6)	116 (85.9)	16 (11.9)	28 (20.7)	66 (48.9)	35 (25.9)	37 (27.4)	135
M694V/E148Q	116 (87.2)	106 (79.7)	26 (19.5)	25 (18.8)	54 (40.6)	23 (17.3)	20 (15)	133
M680I/–	103 (84.4)	107 (87.7)	10 (8.2)	32 (26.2)	58 (47.5)	33 (19)	25 (20.5)	122
M680I/M680I	113 (93.4)	113 (93.4)	35 (28.9)^*^	15 (12.4)	35 (28.9)	10 (8.3)	10 (8.3)	121
M680I/V726A	79 (96.3)	75 (91.5)	22 (26.8)	9 (11)	25 (30.5)	12 (14.6)	7 (8.5)	82
M694V/R761H	58 (93.5)	56 (90.3)	20 (32.3)^*^	9 (14.5)	19 (30.6)	6 (9.7)	5 (8.1)	62
R761H/–	30 (85.7)	28 (80)	6 (17.1)	10 (28.6)^*^	18 (51.4)	9 (25.7)	11 (31.4)^*^	35
E148Q/E148Q	25 (80.6)	25 (80.6)	5 (16.1)	6 (19.4)	14 (45.2)	6 (19.4)	6 (19.4)	31
A744S/–	28 (93.3)	26 (86.7)	7 (23.3)	6 (20)	16 (53.3)^*^	7 (23.3)	9 (21)	30
M680I/E148Q	23 (76.7)	24 (80)	10 (33.3)^*^	5 (16.7)	11 (36.7)	5 (16.7)	8 (26.7)	30
Total (*n*)	2787	2748	654	900	1443	712	705	

*ELP, Exertional leg pain*.

* *Statistically significant (p < 0.001)*.

When patients with and without homozygous or compound heterozygous mutations in exon 10 were compared, it was seen that children carrying homozygous or compound heterozygous mutations on exon 10 had an earlier age of disease onset (4.6 vs. 5.6 years, *p* < 0.001) and a higher number of attacks per year (11.1 vs. 9.6, *p* = 0.001). However, the delay in diagnosis was found to be longer in this group than in the other group (18 vs. 12 months, *p* < 0.001). In addition, it was found that fever (*p* < 0.001), abdominal pain (*p* < 0.001), chest pain (*p* < 0.001), arthritis (*p* < 0.001), and ELE (*p* < 0.001) were more common in children with homozygous or compound heterozygous mutations in exon 10. The comparison of patients carrying homozygous or compound heterozygous mutations in exon 10 and patients carrying other mutations are shown in Table 4. Although 8% of the patients had a family history of amyloidosis, amyloidosis was present in 0.3% (*n* = 11) of the cohort. All patients were diagnosed with kidney biopsy. M694V homozygosity was detected in nine patients who developed amyloidosis. The patients diagnosed with amyloidosis were treated with anti-IL-1 agents.

**Table 4:** The differences between Familial Mediterranean fever patients carrying homozygous or compound heterozygous mutations in exon 10 and patients carrying other mutations.

**Clinical findings**	**Homozygous or compound heterozygous mutations in exon 10 (*n* = 1,864)**	**Other mutations (*n* = 1,590)**	***p*-value**
Age of disease onset^*^	4.6 ± 3.5	5.6 ± 4.0	*p* < 0.001
Number of attacks per year^*^	11.1 ± 10.6	9.6 ± 9.3	*p* = 0.001
Diagnostic delay, median months (min–max)	18 (0–203)	12 (0–192)	*p* < 0.001
Fever, *n* (%)	1,659 (89)	1,335 (84)	*p* < 0.001
Abdominal pain, *n* (%)	1,681 (90.2)	1,365 (85.8)	*p* < 0.001
Chest pain, *n* (%)	487 (26.1)	212 (13.3)	*p* < 0.001
Arthritis, *n* (%)	609 (32.7)	348 (21.9)	*p* < 0.001
Arthralgia, *n* (%)	826 (44.3)	736 (46.3)	*p* = 0.245
Myalgia, *n* (%)	413 (22.2)	380 (23.9)	*p* = 0.225
Erysipelas-like erythema, *n* (%)	325 (17.4)	128 (8.1)	*p* < 0.001
Exertional leg pain, *n* (%)	433 (23.2)	331 (20.8)	*p* = 0.089
Protracted febrile myalgia, *n* (%)	39 (2.1)	15 (0.9)	*p* = 0.007
Constipation	113 (6.1)	87 (5.5)	*p* = 0.459
Diarrhea	140 (7.5)	135 (8.5)	*p* = 0.289
Resistant to colchicine treatment, *n* (%)	138 (7.4)	11 (0.7)	*p* < 0.001

* *Mean ± standard deviation*.

All patients in the cohort were on colchicine therapy, but 149 (4.3%) of them were colchicine-resistant. While exon 10 homozygous or compound heterozygous mutations were detected in 138 (91.6%) of these patients, the most common mutation was M694V homozygous (*n* = 121, 81.2%). Of the patients with colchicine resistance, 92 (61.7%) were girls and there was a significant difference between the two genders in terms of colchicine resistance (*p* = 0.006). Anti-IL 1 agents (Anakinra or Canakinumab) were added to the treatment of all these patients and remission was achieved.

## Discussion

This study investigated the relationship between demographics, clinical features, and genetic outcomes in a large cohort of FMF. In addition to the clinical findings, which are among the classification criteria, the relationship with the genetic results in other common findings was investigated. In addition, the most up-to-date information about the frequency of amyloidosis, which is the most important complication of the disease, is given.

It is well-known that exon 10 homozygous mutations cause more severe disease, and it has been shown that the symptoms of the disease appear earlier in patients with these mutations (8, 22). In our study, it was shown that disease symptoms started earlier in patients with exon 10 homozygous or combined heterozygous mutations.

Consistent with the literature in this study, the most common symptoms were abdominal pain (88.2%) and fever (86.7%). The incidence of fever in FMF has been reported as 82.9–93.1% (8, 20, 23, 24). Cases with afebrile FMF attacks have also been reported (18, 25). IL-1β is probably the main cause of fever, but attention should be paid to coexisting conditions such as microsomal prostaglandin E synthase-1 deficiency in afebrile FMF patients (26, 27). Therefore, additional research is needed to examine the mechanism in afebrile patients with FMF. The frequent occurrence of arthralgia, exertional leg pain, and myalgia, which are not among the classification criteria, suggests that these findings should also be paid attention to. Similar results have been found in the previous large series (8, 20, 28).

More than 300 *MEFV* mutations have been identified to date, and the pathogenic mutations are mostly located in exon 10 such as M694V, M680I, V726A, and M694I. Among these mutations, M694V is the most common with a frequency of 20–65% (3). In this study, the allele frequency was calculated, and the most common mutations were found to be M694V, M680I, V726A, and E148Q, respectively. In line with the literature, the M694V homozygous mutation (28.1%) was identified as the most common variant in our study. To make a genotype-phenotype correlation, patients were divided into those carrying and not carrying homozygous or compound heterozygous exon 10 mutations, which are known to be pathogenic and accepted as confirmatory mutations according to the new classification criteria (17). In this study, it was shown that abdominal pain, fever, chest pain, and arthritis were more common in patients with homozygous or compound heterozygous exon 10 mutations compared to the other group. However, ELE, which is not among the diagnostic criteria, was also found more frequently in patients with homozygous or combined heterozygous exon 10 mutations. It has been shown that this symptom, which is less common than other findings, should be paid attention to and questioned.

Whether the E148Q variant is a disease-causing mutation, or a polymorphism is still a matter of debate. It has been reported that the E148Q variant has unknown pathogenic significance and carrying this variant alone cannot support the diagnosis of FMF (9, 10). E148Q was found to be the fourth most common mutation in our cohort. In a recent study by Tirosh et al. (29), it was reported that the presence of the E148Q variant with the M694V variant was not worsening the clinical phenotype. However, M694V/E148Q mutation was found in three of the patients who did not respond to colchicine in our study. Of course, the pathogenic role of M694V cannot be denied here, but it is clear that the discussions about E148Q are not over yet. Therefore, it should be noted that the diagnosis of FMF is still based on clinical evaluation and genetic analysis can be supportive.

Amyloidosis is still the most serious complication of FMF and only 11 of our patients had amyloidosis. While M694V homozygous mutation was found in nine of the patients who developed amyloidosis, M694V heterozygous mutation was found in one and M694V/M680I mutations were detected in the other patient. This rate (0.3%) was lower than the previous reports regarding amyloidosis (19, 21, 23, 30–32). Akse-Onal et al. (33) evaluated the distribution of amyloidosis frequency by years and they demonstrated a significant decline in the frequency of secondary amyloidosis from 12.1% (1978–1990) to 2% (after 2000; *p* < 0.001). This may be a result of increased awareness of the disease, not delaying the diagnosis, and presence of new treatment options in cases resistant to colchicine. Proven anti-IL-1 treatments were used in these patients and clinical response was obtained in accordance with the literature (34, 35).

Although this study is limited in its retrospective design, to our knowledge it is the largest FMF series of children evaluating the phenotype-genotype correlation as a real-life data. In this study, it has been demonstrated that exon 10 mutations, particularly the M694V homozygous mutation, are important in disease severity and outcome. Although E148Q is considered as a polymorphism in some populations, it was identified as a disease-causing mutation in our cohort. Secondary amyloidosis still occurs in adults, but is extremely rare among children, probably due to increased awareness, tight control, and availability of anti-IL1 agents in colchicine-resistant cases.

## Data Availability Statement

The original contributions presented in the study are included in the article/supplementary material, further inquiries can be directed to the corresponding author.

## Ethics Statement

The studies involving human participants were reviewed and approved by TC Saglik Bakanligi Istanbul Saglik Bilimleri Üniversitesi Ümraniye Egitim ve Araştirma Hastanesi Klinik Araştirmalar Etik Kurulu. Written informed consent to participate in this study was provided by the participants' legal guardian/next of kin.

## Author Contributions

KÖ, NA, and BS designed the study. KÖ, TC, EB, HS, GY, FÇ, FGD, AT, SK, SÖ, FD, and MÇ collected and analyzed data. KÖ, HS, NA, and BS wrote the manuscript. All authors have read and approved the final manuscript.

## Conflict of Interest

The authors declare that the research was conducted in the absence of any commercial or financial relationships that could be construed as a potential conflict of interest. The reviewer EB declared a past co-authorship with several of the authors KÖ, EB, HS, GY, FÇ, SK, SO, FD, MÇ, NA, and BS and reviewer ES declared a past collaboration with several of the authors HS, SK, SO, FD, NA, and BS to the handling editor at the time of the review.

## Publisher's Note

All claims expressed in this article are solely those of the authors and do not necessarily represent those of their affiliated organizations, or those of the publisher, the editors and the reviewers. Any product that may be evaluated in this article, or claim that may be made by its manufacturer, is not guaranteed or endorsed by the publisher.
